# Synthesis and Crystal Structure of Diaqua(1,10-Phenanthroline-*N,N′*)(Thiosulfato-O,S)Manganese(II). Biological Properties.

**DOI:** 10.1155/MBD.1998.305

**Published:** 1998

**Authors:** Maria Brezeanu, Mikaela Badea, Georges Morgant, Bernard Viossat, Sylvie Bouttier, Jacky Fourniat, Dana Marinescu, Dung Nguyen Huy

**Affiliations:** 1 Laboratory of Inorganic Chemistry Faculty of Chemistry University of Bucharest Romania; 2 Laboratoir de Cristallochimie Bioinorganique Faculté de Pharmace Paris Xl Châtenay-Malabry paris France; 3 Laboratoire de Biochimie Hôpital Armand Trousseau Paris France; 4 Laboratoire de Chimie Générale Faculté de Médecine et de Pharmacie Poitiers France; 5 Laboratoire de Microbiologie Industrielle Faculté de Pharmace Paris Xl Châtenay-Malabry paris France; 6 Laboratoire de Cristallochimie Bioinorganique Faculté de Pharmace Paris Xl, 5 rue Jean-Baptiste Clément Châtenay-Malabry, Cedex Paris 92290 France

## Abstract

The synthesis of diaqua(1,10-phenanthroline-*N,N′*)(thiosulfato-O,S)manganese(ll) [Mn(phen)(S_2_O_3_)(H_2_O)_2_] was investigated. Its structure was determined by single crystal X-ray diffraction from 2418 reflections (I > 3 σ(I)) to a final value of R = 0.047 and Rw = 0.054. Crystal data are as follows : space group P_2__1_; a = 10.356(3), b = 7.097(3), c = 20.316(2) Å, β = 94.29(2)°, V = 1489.1(8) , Å^3^, Z = 2. There are two independent title compounds in the asymetric unit. Each manganese atom has a distorted octahedral Mn(SO)N_2_O_2_ geometry with the S and O atoms (from two neighbouring thiosulfate ligands) mutually *trans*, two N atoms from the 1,10-phenanthroline ligand and two water oxygen. The thiosulfate group behaves as a bridging ligand, connecting, through sulfur and oxygen, Mn atoms related by the binary **b** translation, thus forming infinite chains running parallel to this axis. Infrared and electronic spectra are reported.


### SYNTHESIS AND CRYSTAL STRUCTURE OF DIAQUA(1,10-PHENANTHROLINE-*N,N'*)(THIOSULFATO-O,S)MANGANESE(II). BIOLOGICAL PROPERTIES.

Maria Brezeanu[1], Mikaela Badea[1], Georges Morgant[2,3], Bernard Viossat[4], Sylvie Bouttier[5], Jacky Fourniat[5], Dana Marinescu[1] and Dung Nguyen Huy[2]*

[1] Laboratory of Inorganic Chemistry, Faculty of Chemistry, University of Bucharest, Romania

[2] Laboratoire de Cristallochimie Bioinorganique, Faculté de Pharmacie Paris XI, Châtenay-Malabry, France

[3] Laboratoire de Biochimie, Hôpital Armand Trousseau, Paris , France

[4] Laboratoire de Chimie Générale, Faculté de Médecine et de Pharmacie, Poitiers, France

[5] Laboratoire de Microbiologie Industrielle, Faculté de Pharmacie Paris XI, Châtenay-Malabry, France

* Correspondence and reprint requests to Nguyen Huy Dung, Laboratoire de Cristallochimie Bioinorganique, Faculté de Pharmacie Paris XI, 5, rue Jean-Baptiste Clément, 92290 Châtenay-Malabry, Cedex, France. E-mail : Morgant@cep.u-psud.fr

**Abstract**

The synthesis of diaqua(1,10-phenanthroline-*N,N'*)(thiosulfato-O,S)manganese(II) $[Mn(phen)(S_2O_3)(H_2O)_2]$ was investigated. Its structure was determined by single crystal X-ray diffraction from 2418 reflections ($I > 3\ \sigma(I)$) to a final value of R = 0.047 and Rw = 0.054. Crystal data are as follows : space group $P2_1$ ; a = 10.356(3), b = 7.097(3), c = 20.316(2) Å, $\beta$ = 94.29(2)°, V = 1489.1(8) $Å^3$, Z = 2. There are two independent title compounds in the asymetric unit. Each manganese atom has a distorted octahedral $Mn(SO)N_2O_2$ geometry with the S and O atoms (from two neighbouring thiosulfate ligands) mutually *trans*, two N atoms from the 1,10-phenanthroline ligand and two water oxygen. The thiosulfate group behaves as a bridging ligand, connecting, through sulfur and oxygen, Mn atoms related by the binary **b** translation, thus forming infinite chains running parallel to this axis. Infrared and electronic spectra are reported.

### 1. Introduction

The molecule 1,10-phenanthroline (phen) and its derivatives are well known to exhibit antifungal, antiviral and mycoplasmal activities [1]. Their cytotoxicity might be due to chelation of transition metals such as copper or iron in test media. Complexation with some divalent transition metals enhances these biological activities. Thus, antiviral activity was found for divalent transition metals with substituted phenanthrolines in the following order of activity Cd > Cu >> Zn >> Mn > Fe > Co > Ni > Ru [2]. Moreover, these complexes are used as DNA intercalating agents [3] and have been found useful for examining distinctive conformations along the DNA helix [4]. Recently, ternary complexes of Cu(II) with 3,5-diisopropylsalicylate and substituted phenanthrolines have been synthetised : among them, the most potent was bis(diisopropylsalicylato)(2,9-dimethylphenanthroline)copper(II) which exhibits cytotoxicity comparable with cisplatin $(PtCl_2(NH_3)_2)$, an anticancer drug. It was suggested that incorporation of a phen ligand in this former ternary Cu(II) complex may favour DNA intercalation [5].

Moreover, in the dinuclear complex $[Rh_2(OAc)_4(H_2O)_2]$, cytostatic activity was found to be enhanced when phen (or one of its derivatives) was incorporated in dimeric cationic rhodium(II) complexes such as $[Rh_2(RCOO)_2phen_2(H_2O)_2]^{2+}$ where R = $CH_3$, $CH_3CHOH$, $C_6H_5CHOH$ and phen = 1,10-phenanthroline or 4,7-diphenyl-1,10-phenanthroline. Biological activities of these complexes are similar, for both KB cell line carcinoma and synchronous culture of *chlorella vulgaris* [6, 7].

Considerable attention has been focused on the bioinorganic chemistry of manganese and especially on metalloenzymes with multinuclear Mn centres [8]. Recently, the synthesis and X-ray crystal structure of the $Mn^{II,II}$ complex double salt $[Mn_2(\eta^1\eta^1\mu_2\text{-oda})(phen)_4(H_2O)_2]$ $[Mn_2(\eta^1\eta^1\mu_2\text{-oda})(phen)_4(\eta^1\text{-oda})_2].4(H_2O)$ (oda $H_2$ = octanedioic acid) were reported together with its catalytic activity towards the disproportionation of $H_2O_2$ [9].

To our knowledge, very few manganese thiosulfate complexes have been studied by X-ray diffraction. An X-ray study of these complexes would not only give the coordination around the metal atom but should also define the situation of the thiosulfate group, which could in theory coordinate as a monodentate ligand, a bidentate ligand, or as a bridging ligand between different metal atoms.

### 2. Results and discussion.

### 2.1. Description of [Mn(phen)($S_2O_3$)($H_2O$)$_2$] structure.

Distances and angles in the coordination sphere are shown in Tables 1 and 2. The asymmetric unit consists of two independent title molecules, called hereafter (a) and (b), being complexes of Mn(1) and Mn(2), respectively. Their structures are very similar to each other. To get atom numbers for the second molecule, add 1 to Mn and 20 to the other atom numbers. The Mn(1) atom has a distorted octahedral trans Mn(SO)$N_2O_2$ geometry, the basal plane being formed by two N atoms (N(1) and N(10)) from the 1,10-phenanthroline and two water oxygens, labeled O(4) and O(5). The two axial positions are occupied by one sulfur atom S(2) from the thiosulfate ligand and one oxygen atom O($1^i$) from a neighbouring thiosulfate (symmetry code i : x, y, 1 - z). Thus the thiosulfate anion behaves as a bidentate ligand. The same coordination was found for the Mn(2) atom. The bond distances Mn(1) - S(2) (2.631(2) Å) and (Mn(2) - S(22) (2.648(2) Å) are comparable with those found in (1,10-phenanthroline-*N,N'*)-bis(4,6-dimethylpyrimidine-2-thiolato-N,S)-manganese(II) [2.591 and 2.594 Å] [10]. The Mn(1) - O($1^i$) distance [2.203(6) Å] is significantly longer than the other metal-oxygen distances, Mn(1) - O(4) ($H_2O$) and Mn(1) - O(5) ($H_2O$) [2.147(5) and 2.140(6) Å]. These latter values are similar to those found in diaqua-bis(1,10-phenanthroline-*N,N'*)manganese(II) bis(saccharinate) monohydrate (2.128 and 2.142 Å for Mn - O ($H_2O$) distances) [11] or in [$Mn_2(\eta^1\eta^1\mu_2$-oda)(phen)$_4$($H_2O$)$_2$] [$Mn_2(\eta^1\eta^1\mu_2$-oda)(phen)$_4$($\eta^1$-oda)$_2$].4($H_2O$) with Mn - O ($H_2O$) [2.123(4) Å] and where $oda^{2-}$ is the anion of the octanedioic acid [9]. However, in the molecule (b) the apical distance Mn(2) - O($21^i$) is equivalent to Mn(2) - O(24) ($H_2O$) which is significantly longer (2.185(5) Å) than the other Mn(2) - O(25) ($H_2O$) distance (2.130(5) Å).

Table 1 : Selected interatomic distances (Å). E.s.d's in parentheses refer to the last significant digit.

| | | | | | | | |
|---|---|---|---|---|---|---|---|
| Mn(1) | - | S(2) | 2.631(2) | Mn(2) | - | S(22) | 2.648(2) |
| Mn(1) | - | O($1^i$) | 2.203(6) | Mn(2) | - | O($21^i$) | 2.184(6) |
| Mn(1) | - | O(4) | 2.147(5) | Mn(2) | - | O(24) | 2.185(5) |
| Mn(1) | - | O(5) | 2.140(6) | Mn(2) | - | O(25) | 2.130(5) |
| Mn(1) | - | N(1) | 2.281(6) | Mn(2) | - | N(21) | 2.265(6) |
| Mn(1) | - | N(10) | 2.257(6) | Mn(2) | - | N(30) | 2.272(6) |
| S(1) | - | S(2) | 2.006(3) | S(21) | - | S(22) | 2.005(3) |
| S(1) | - | O(1) | 1.475(6) | S(21) | - | O(21) | 1.458(6) |
| S(1) | - | O(2) | 1.461(6) | S(21) | - | O(22) | 1.474(6) |
| S(1) | - | O(3) | 1.479(6) | S(21) | - | O(23) | 1.465(6) |

Symmetry code : i : x, y - 1, z

The two 1,10-phenanthroline ligands are bidentate. The Mn(1) - N(1) and Mn(1) - N(10) distances of 2.281(6) to 2.257(6) Å (or Mn(2) - N(21) and Mn(2) - N(30) distances of 2.265(6) to 2.272(6) Å in complex (b)) are in the range of 2.421 to 2.301 Å found in diaqua-bis(1,10-phenanthroline-*N,N'*)-manganese(II) bis(saccharinate) monohydrate [11]. The N(1) - Mn(1) - N(10) and N(21) - Mn(2) -N(30) angles (73.2(2)°) are close to those found in the latter complex. The phen ligands are almost planar (maximum deviation from the least-squares plane is 0.063 Å in complex (a) and 0.054 Å in complex (b)). In complex (a), phen makes an interplanar angle of 170.8° with the four atoms of coordination equatorial plane N(1), N(10), O(4) and O(5) from which the Mn(1) atom is displaced by 0.023 Å in the direction of the apical O($1^i$) atom. On the other side, phen makes an interplanar angle of 4.55° with the equatorial plane N(21), N(30), O(24) and O(25) from which the Mn(2) atom is displaced by -0.031 Å towards the S(22) atom. The out-of-plane displacements of Mn(1) from the two pyridine rings are 0.177 and -0.153 Å (0.090 Å and -0.099 Å in complex (b)). The dimensions of the 1,10-phenanthroline molecule are not significantly different from those observed for the free ligand [12] or in complexes containing them [13].

In the title complex, values for S - S 2.006(3) è and S - O bond distances in the range of 1.479 to 1.458 Å agree fairly well with those observed in ionic magnesium thiosulfate $MgS_2O_3$. 6 $H_2O$ [14] in which the anion is uncomplexed and with mean values for S - S and S - O distances 2.013 and 1.467 Å or in bis(ethylenethiourea) zinc(II) thiosulfate [15] where thiosulfate is also bridging ligand (mean values for S - S and S - O distances 2.025 and 1.468 Å). In [Cd(dmph)($S_2O_3$)] (dmph =

dimethylphenanthroline), the $S_2O_3$ group presents an unusual type of coordination acting both as a bridging and bidentate ligand [16]. Moreover, in [Cd(bipy)($S_2O_3$)] (bipy = 2,2'-bipyridine), the bridging thiosulfates bind metal centres through two different sequences Cd1 - S - Cd1' and Cd1 - O1' - S2' - S1' - Cd1', thus defining a six-membered ring [17]. In the title compound, thiosulfate bridging ligands connect, through sulfur and oxygen, Mn atoms which are related by the binary **b** axis translation, giving rise to polymeric chains running parallel to this axis. This is illustrated in Figure 1.

Table 2 : Selected bond angles (°). E.s.d's in parentheses refer to the last significant digit.

| | | | | | | | |
|---|---|---|---|---|---|---|---|
| S(2) | Mn(1) | O(1$^i$) | 177.7(1) | S(22) | Mn(2) | O(21$^i$) | 176.6(2) |
| S(2) | Mn(1) | O(4) | 92.0(2) | S(22) | Mn(2) | O(24) | 90.5(2) |
| O(1$^i$) | Mn(1) | O(4) | 88.3(2) | O(21$^i$) | Mn(2) | O(24) | 86.9(2) |
| S(2) | Mn(1) | O(5) | 91.8(2) | S(22) | Mn(2) | O(25) | 91.6(2) |
| O(1$^i$) | Mn(1) | O(5) | 85.8(2) | O(21$^i$) | Mn(2) | O(25) | 90.8(2) |
| O(4) | Mn(1) | O(5) | 97.2(3) | O(24) | Mn(2) | O(25) | 95.5(2) |
| S(2) | Mn(1) | N(1) | 81.1(2) | S(22) | Mn(2) | N(21) | 85.9(2) |
| O(1$^i$) | Mn(1) | N(1) | 99.1(3) | O(21$^i$) | Mn(2) | N(21) | 92.2(3) |
| O(4) | Mn(1) | N(1) | 165.9(2) | O(24) | Mn(2) | N(21) | 95.0(2) |
| O(5) | Mn(1) | N(1) | 95.3(3) | O(25) | Mn(2) | N(21) | 169.2(3) |
| S(2) | Mn(1) | N(10) | 91.6(2) | S(22) | Mn(2) | N(30) | 95.0(2) |
| O(1$^i$) | Mn(1) | N(10) | 90.7(2) | O(21$^i$) | Mn(2) | N(30) | 87.1(3) |
| O(4) | Mn(1) | N(10) | 94.9(2) | O(24) | Mn(2) | N(30) | 166.6(2) |
| O(5) | Mn(1) | N(10) | 167.3(3) | O(25) | Mn(2) | N(30) | 96.5(3) |
| N(1) | Mn(1) | N(10) | 73.2(2) | N(21) | Mn(2) | N(30) | 73.3(2) |
| S(2) | S(1) | O(1) | 108.5(3) | S(22) | S(21) | O(21) | 108.7(3) |
| S(2) | S(1) | O(2) | 107.3(3) | S(22) | S(21) | O(22) | 108.7(3) |
| O(1$^i$) | S(1) | O(2) | 110.9(3) | O(21) | S(21) | O(22) | 108.9(4) |
| S(2) | S(1) | O(3) | 110.0(3) | S(22) | S(21) | O(23) | 108.8(2) |
| O(1) | S(1) | O(3) | 109.7(3) | O(21) | S(21) | O(23) | 111.0(3) |
| O(2) | S(1) | O(3) | 110.5(4) | O(22) | S(21) | O(23) | 110.8(3) |
| Mn(1) | S(2) | S(1) | 115.6(1) | Mn(2) | S(22) | S(21) | 114.6(1) |
| Mn(1) | O(1) | S(1) | 139.3(3) | Mn(2) | O(21) | S(21) | 141.2(3) |
| Mn(1) | N(1) | C(2) | 127.5(6) | Mn(2) | N(21) | C(22) | 126.6(6) |
| Mn(1) | N(1) | C(13) | 115.5(4) | Mn(2) | N(21) | C(33) | 115.0(5) |
| C(2) | N(1) | C(13) | 116.9(7) | C(22) | N(21) | C(33) | 118.4(7) |
| Mn(1) | N(10) | C(9) | 126.5(5) | Mn(2) | N(30) | C(29) | 126.9(7) |
| Mn(1) | N(10) | C(11) | 115.8(4) | Mn(2) | N(30) | C(31) | 114.5(5) |
| C(9) | N(10) | C(11) | 117.6(6) | C(29) | N(30) | C(31) | 118.6(8) |

It is noteworthy that the S(22) - O(21$^i$) direction in the $S_2O_3$ bridging ligand is nearly perpendicular to the mean plane of the phen molecule b (83.4°),while S(2) - O(1$^i$) makes an angle of 75.4°with phen molecule a.

Fig 1 shows a perspective view of the tittle molecule in which probable hydrogen bonding is represented by dotted lines. Hydrogen atoms attached to water molecules could not be located by a difference FOURIER synthesis, but it is suggested that water molecules (via hydrogens attached to O(4) and O(5) or O(24) and O(25)) might be engaged in hydrogen bonding with O atoms of the thiosulfate groups, thus defining intramolecular bondings within the infinite chains of the Mn(1).......Mn(1$^i$)..... or Mn(2).......Mn(2$^i$)..... atoms and intermolecular bonding between the two different chains.

### 2.2. Infrared and electronic spectra.

The most important aspect of the IR spectrum of [Mn(phen)($S_2O_3$)($H_2O$)$_2$] concerns the bands of the thiosulfate anion.

It is known that the uncoordinated thiosulfate anion shows IR bands which are assigned to the vibrations : $\nu_s(SO_3)$, $\nu$(S-S), $\delta_{as}(SO_3)$, $\nu_{as}(SO_3)$, $\delta_s(SO_3)$ and $\rho_r(SO_3)$ at 995, 446, 669, 1123, 541 and 335 $cm^{-1}$ respectively [18]

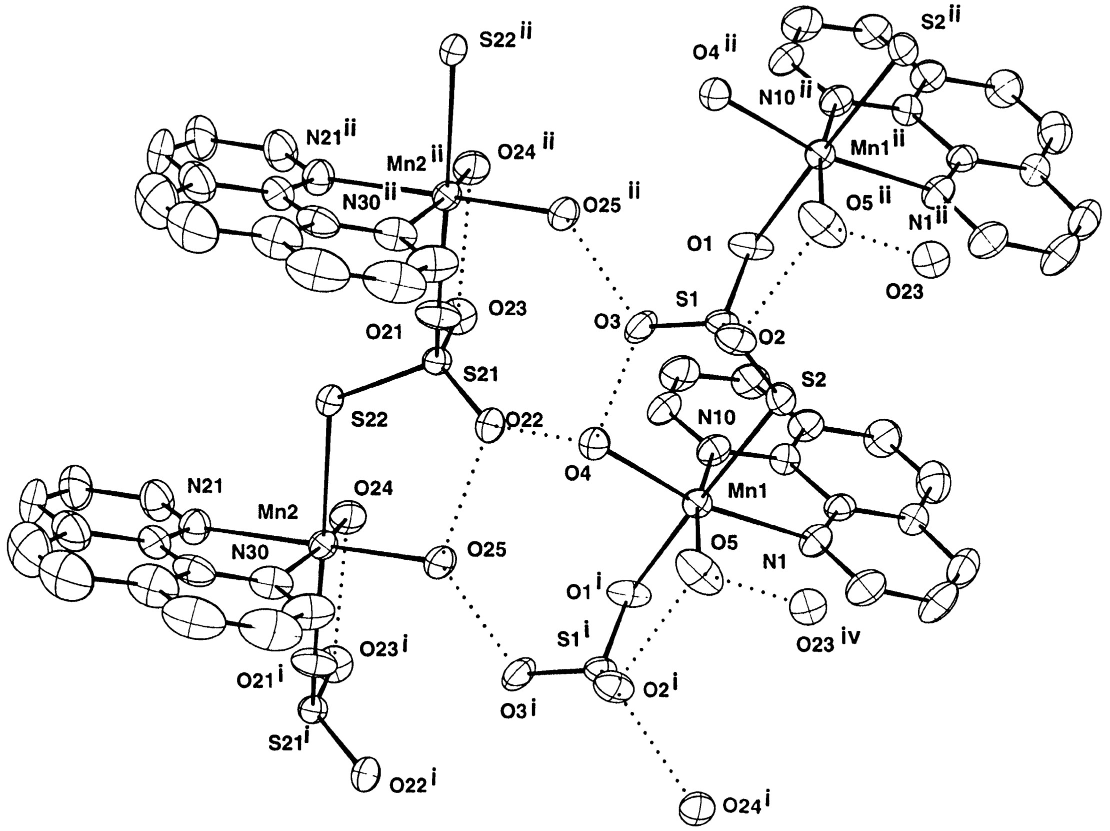

Figure 1 : Molecular structure of diaqua(1,10-phenanthroline-*N,N'*)(thiosulfato-O,S)manganese(II). Displacement ellipsoïds are plotted at 50% probability level. Hydrogen atoms are omitted for clarity. Symmetry code : i = x, y - 1, z ; ii = x, y + 1 ; z, iii = x - 1, y + 1, z ; iv = x - 1, y, z ;

As a bridging ligand, the thiosulfate group shows the following changes in the stretching mode : a shift to lower energy of ν(S-S) and $\nu_s(SO_3)$ and a splitting of $\nu_{as}(SO_3)$ into two components due to the interactions M-O and M-S.

In fact, the IR spectrum of $[Mn(phen)(S_2O_3)(H_2O)_2]$ is consistent with this coordination mode, namely the occurrence of bands assigned to ν(S-S) at 420, $\nu_s(SO_3)$ at 970 and $\nu_{as}(SO_3)$ splitted in two components at 1100 and 1150 $cm^{-1}$ (the component at 1100 $cm^{-1}$ is due to the Mn-O and the second, to the Mn-S interactions).

Further absorption bands at 630, 720, 850, 960, 1000, 1420, 1440, 1520 and 1535 $cm^{-1}$ are assigned to the vibrational modes of phenanthroline, which indicate coordination of nitrogen atoms to the metal atom, and there is a band at 520 $cm^{-1}$ ($\rho_w H_2O$) consistent with the coordinated water molecules.

The appearance of a metal-oxygen band at ca. 380 $cm^{-1}$ further supports this view.

Thus, it appears that the manganese atom is coordinated by the N atoms from 1,10-phenanthroline ligand, one sulfur atom from one thiosulfate group, one oxygen atom from one neighbouring $S_2O_3$ group and water oxygen. The thiosulfate group acts as bridging ligand through oxygen and sulfur atoms.

The diffuse reflectance spectrum of complex $[Mn(phen)(S_2O_3)(H_2O)_2]$ is consistent with a 6-coordinated high-spin Mn(II), for which all the d-d transitions are spin-forbidden. An intense MLCT band due to the $e_g \rightarrow \pi^*$ electronic transfer is observed at 24.3 kK [19].

The spectrum was processed automatically, giving two low intensity bands assigned to the spin forbidden transitions to the $^4T_2(D)$ (26.3 kK) and $^4T_1(G)$ (19.5 kK) terms [19]. The assignments are based on approximate $O_h$ symmetry (the distortion appearing as a consequence of the different nature of the ligands) the chromophore being $[Mn(II)N_2O_3S]$.

2.3. Biological assays.

The three components in the complex : $S_2O_3^{2-}$, $Mn^{II}$ and 1,10-phenanthroline were tested separately. It is well known that $S_2O_3^{2-}$ usually used as neutralizing agent towards some antiseptic oxidizing agents (such as sodium hypochlorite) at the concentration of 0.5% w/V does not exhibit antimicrobial activity [20]. Moreover, it was verified that $Mn^{II}$ (at $10^{-3}$ M) does not exhibit antibacterial activity towards the tested microorganisms (data not shown). Finally, it was demonstrated that the ternary complex does not modify significantly the antibacterial and antifungal activities versus 1,10-phenanthroline alone, as shown in the table below :

| | Complex - MIC (M) | 1,10-phenanthroline - MIC (M) |
|---|---|---|
| Staphylococcus aureus | 7.80 $10^{-5}$ | 6.25 $10^{--4}$ |
| Escherichia coli | 1.56 $10^{-4}$ | 7.80 $10^{-5}$ |
| Neisseria subflava | 7.80 $10^{-5}$ | 7.80 $10^{-5}$ |
| Candida albicans | 7.80 $10^{-5}$ | 7.80 $10^{-5}$ |

These results are in agreement with those obtained by studying the toxicity of 1,10-phenanthroline and its reversal by divalent transition metal ions, specially $Mn^{II}$ [21].

### 3. Experimental

3.1. Materials

$Mn(CH_3COO)_2.4H_2O$, $Na_2S_2O_3.5H_2O$ and 1,10-phenanthroline were purchased from commercial sources and used as received (p.a. grade). Elemental analyses (C, H, N, S, Mn) were performed by usual microanalytical methods.

Microbial strains

*Staphylococcus aureus* CIP 4.83, *Escherichia coli* CIP 54.127, *Neisseria subflava* CIP 52.180 and *Candida albicans* CIP 48.72 were used as test organisms. Bacterial strains were grown on Tryptic Soy Agar (Merck) at 37°C for 24 h. Yeast strain was grown on Sabouraud agar (Bio-Mérieux) at 30 °C for 48 h. Microbial strains were dispersed in a diluent containing Tryptone (Difco) (1 g/L) and sodium chloride (8.5 g/L) in distilled water. The suspensions were adjusted to 1-3.$10^8$ colony-forming units per mL (CFU/ml).

3.2. Synthesis of $[Mn(phen)(S_2O_3)(H_2O)_2]$

An aqueous solution containing in 125 mL $H_2O$, $Mn(CH_3COO)_2.4H_2O$ (4 mmol), $Na_2S_2O_3.5H_2O$ (8mmol) and 1,10-phenanthroline (4 mmol) was boiled during two hours.

The orange-coloured product was sparingly soluble in hot solution, and was filtered off and the yellowish-green filtrat allowed to evaporate at room temperature.

The pale yellow crystals separated after a few days; they were filtered off and washed with water and ethanol. (Found : C = 37.95, H = 3.30, N = 7.97, S = 17.25, Mn = 14.98%. Calculated for $MnC_{12}H_{12}N_2S_2O_5$ : C = 37.60, H = 3.13, N = 7.31, S = 16.71, Mn = 14.35%).

3.3. X-ray structure determination

Data were collected at 291 K on an ENRAF-NONIUS CAD4 diffractometer. The final unit cell parameters were obtained by least-squares refinement of 25 reflections. Crystal decay was monitored by measuring three intensity control reflections every two hours. Only statistical fluctuations were observed in the intensity monitors over the course of data collection.

The structure was solved by direct methods (SIR92) [22] and refined by least-squares procedures on Fobs. H atoms (excepted those attached to water molecules) were introduced in calculatated in idealized positions (d (C - H) = 0.96 Å) and their atomic coordinates were recalculated after each cycle. They were given isotropic thermal parameters 20% higher than those of the carbon to which they were attached. Coordinates of the H atoms attached to O atoms could not be located on difference Fourier maps. Least-squares refinements were performed by minimizing the function $\Sigma w(|F_o|-|F_c|)^2$, where $F_o$ and $F_c$ are the observed and calculated structure factors. The weighting scheme used in the last refinement cycles was $w = w'[1-(\Delta F/6\sigma(F_o)^2]^2$ where $w' = 1/\Sigma_1^n A_rT_r(x)$ with 3 coefficients $A_r$ for the Chebyshev polynomial $A_rT_r(x)$ where x was $F_c/F_c(max)$ [23]. Models reached convergence with $R = \Sigma(||F_o|-|F_c||)/\Sigma(|F_o|)$ and $Rw = [\Sigma w(|F_o|-|F_c|)^2/\Sigma w(F_o)^2]^{1/2}$, having values listed in Table 3. Criteria for a satisfactory complete analysis were ratios of rms shift to standard deviation less than 0.1 and no significant features in final difference maps. Details of data collection and refinement are given in Table 4.

Table 3: Crystal data for the title compound.

| | |
|---|---|
| **Crystal Parameters** | |
| compound | $C_{12}H_{12}N_2O_5S_2Mn$ |
| fw(g) | 383.29 |
| shape (colour) | parallelepiped (colourless) |
| size, mm | 0.080, 0.200, 0.430 |
| crystal system | monoclinic |
| space group | $P2_1$ |
| a, Å | 10.356(3) |
| b, Å | 7.097(3) |
| c, Å | 20.316(2) |
| β, ° | 94.29(2) |
| V, $Å^3$ | 1489.1(8) |
| Z | 2 |
| F(000) | 766.22 |
| ρ (calcd), $g.cm^{-3}$ | 1.71 |
| μ (MoKα), $cm^{-1}$ | 11.43 |
| **Data collection** | |
| Diffractometer | Enraf-Nonius CAD4 |
| monochromator | graphite |
| radiation, Å | MoKα (λ = 0.71073) |
| Scan mode | ω - 2θ |
| temperature, K | 291 |
| 2θ range, deg | 4.0< 2θ <48 |
| Absorption correction | DIFABS |
| T min | 0.99 |
| T max | 1.00 |
| no. of rflns collected | 4190 |
| no. of unique rflns | 4190 |
| reflections used | 2418(I>3σ(I)) |
| **Refinement** | |
| R / $R_W$ | 0.047 / 0.054 |
| Weighting Scheme | Chebyshev |
| Coefficient Ar | 2.24,1.09, 1.47 |
| GOF | 1.57 |
| $(\Delta/\sigma)_{max}$ | 0.04 |
| $\Delta\rho_{min}/\Delta\rho_{max}$ (e. $Å^{-3}$) | -0.477/0.475 |
| Number of parameters | 464 |

Calculations were performed with a PC CRYSTALS package program [24]. The structure was drawn using CAMERON [25]. The atomic scattering factors were taken from International Tables for X-ray Crystallography [26]. Fractional atomic coordinates and equivalent thermal parameters for all atoms (except H attached to carbon atoms), are listed in Table 4.

Anisotropic thermal parameters for non-hydrogen atoms and atomic coordinates for H atoms have been deposited at the Cambridge Crystallographic Data Center.

3.4. Spectroscopic techniques

The infrared spectrum was taken on a BIORAD FTS 135 spectrophotometer as KBr pellets in the 4000-300 $cm^{-1}$ range and the diffuse reflectance electronic spectrum with a UNICAM UV/VIS spectrometer UV 4 using MgO as standard.

Table 5 : Fractional atomic coordinates and equivalent isotropic thermal parameter U(eq). E.s.d's in parentheses refer to the last significant digit
U(eq) is defined as the cube root of the product of the principal axes.

| Atom | x/a | y/b | z/c | U(eq) |
|---|---|---|---|---|
| Mn(1) | 0.25382(9) | 0.2500 | 0.32916(5) | 0.0267 |
| S(1) | 0.2376(1) | 0.7853(3) | 0.27825(9) | 0.0263 |
| S(2) | 0.1911(2) | 0.6039(3) | 0.3484(1) | 0.0362 |
| O(1) | 0.2988(5) | 0.9522(8) | 0.3106(3) | 0.0338 |
| O(2) | 0.1176(5) | 0.8367(9) | 0.2401(3) | 0.0379 |
| O(3) | 0.3289(5) | 0.6956(9) | 0.2353(3) | 0.0373 |
| O(4) | 0.3696(5) | 0.3245(8) | 0.2498(3) | 0.0355 |
| O(5) | 0.0770(5) | 0.200(1) | 0.2704(4) | 0.0478 |
| N(1) | 0.1657(5) | 0.218(1) | 0.4281(3) | 0.0330 |
| N(10) | 0.4188(5) | 0.266(1) | 0.4081(3) | 0.0310 |
| C(2) | 0.0406(7) | 0.187(1) | 0.4386(5) | 0.0441 |
| C(3) | -0.0040(9) | 0.183(2) | 0.5009(6) | 0.0467 |
| C(4) | 0.0762(8) | 0.215(1) | 0.5543(5) | 0.0437 |
| C(5) | 0.3029(9) | 0.273(2) | 0.6011(4) | 0.0470 |
| C(6) | 0.4280(8) | 0.295(2) | 0.5906(4) | 0.0436 |
| C(7) | 0.6050(7) | 0.311(1) | 0.5130(4) | 0.0372 |
| C(8) | 0.6388(7) | 0.304(2) | 0.4498(5) | 0.0425 |
| C(9) | 0.5422(7) | 0.283(1) | 0.3986(4) | 0.0351 |
| C(11) | 0.3846(6) | 0.270(1) | 0.4712(3) | 0.0286 |
| C(12) | 0.4740(7) | 0.293(1) | 0.5255(4) | 0.0338 |
| C(13) | 0.2482(6) | 0.246(1) | 0.4821(3) | 0.0277 |
| C(14) | 0.2089(7) | 0.245(1) | 0.5471(4) | 0.0364 |
| Mn(2) | 0.7336(1) | -0.2724(2) | 0.17413(6) | 0.0274 |
| S(21) | 0.7275(1) | 0.2632(3) | 0.22299(9) | 0.0261 |
| S(22) | 0.8069(2) | 0.0825(3) | 0.1618(1) | 0.0328 |
| O(21) | 0.6851(5) | 0.4305(8) | 0.1857(3) | 0.0352 |
| O(22) | 0.6137(5) | 0.1729(9) | 0.2491(3) | 0.0381 |
| O(23) | 0.8243(5) | 0.3115(9) | 0.2767(3) | 0.0377 |
| O(24) | 0.8739(5) | -0.3088(9) | 0.2590(3) | 0.0379 |
| O(25) | 0.5772(5) | -0.191(1) | 0.2303(4) | 0.0429 |
| N(21) | 0.8757(6) | -0.339(1) | 0.0979(3) | 0.0365 |
| N(30) | 0.6176(6) | -0.284(1) | 0.0748(3) | 0.0371 |
| C(22) | 1.0022(8) | -0.369(1) | 0.1093(5) | 0.0440 |
| C(23) | 1.084(1) | -0.403(2) | 0.0593(6) | 0.0547 |
| C(24) | 1.037(1) | -0.404(2) | -0.0032(6) | 0.0529 |
| C(25) | 0.843(2) | -0.381(2) | -0.0844(5) | 0.0709 |
| C(26) | 0.719(2) | -0.352(2) | -0.0968(5) | 0.0657 |
| C(27) | 0.502(1) | -0.290(2) | -0.0525(7) | 0.0672 |
| C(28) | 0.429(1) | -0.263(2) | -0.0008(8) | 0.0600 |
| C(29) | 0.4905(8) | -0.262(2) | 0.0635(6) | 0.0519 |
| C(31) | 0.6897(9) | -0.310(1) | 0.0227(4) | 0.0397 |
| C(32) | 0.635(1) | -0.310(2) | -0.0428(5) | 0.0559 |
| C(33) | 0.8243(8) | -0.343(1) | 0.0348(4) | 0.0388 |
| C(34) | 0.903(1) | -0.376(2) | -0.0181(5) | 0.0514 |

3.5. Minimum inhibitory concentration determinations.

Minimum inhibitory concentration (MIC) were determined by a broth dilution technique (serial twofold dilution). A solution of the product was prepared in a $H_2O$/DMSO mixture (50/50 V/V) to obtain a concentration of 5 $10^{-2}$ M. The test volume in each vial was 1 mL. Mueller-Hinton broth (BioMérieux) for bacteria and Sabouraud broth (BioMérieux) for yeast were inoculated to obtain approximately 1-3.$10^5$ CFU/ml. Vials were incubated at 37°C for 24 h for bacteria and 30°C for 24 h for yeast. The MIC was determined as the lowest concentration of product yielding no visible growth.

### References

1 R. MacLeod, *J. Biol. Chem.*, **197**, 751 (1952).
2 A. Shulman and D. O. White, *Chem. Biol. Interact.*, **6**, 407 (1973).
3 A. Shulman, G. M. Laycock and T. R. Bradley, *Chem. Biol. Inter.*, **16**, 89 (1977)
4 J. K. Barton, *Science*, **233**, 727 (1986).
5 J. D. Ranford, P. J. Salder and D. A. Tocher, *J. Chem. Soc. Dalton Trans.*, 3393 (1993).
6 F. P. Pruchnik, G. Kluczewska, A. Wilczok, U. Mazurek and T. Wilczok, *J. Inorg. Biochem.*, **65**, 25 (1997).
7 F. P. Pruchnik and D. Dus, *J. Inorg. Biochem.*, **61**, 55 (1996).
8 E. J. Larson and V. L. Pecoraro in *Manganese Redox Enzymes*, ed V. L. Pecoraro, VCH, New York, ch. 1 (1992).
9 M. T. Casey, M. McCann, M. Devereux. M. Curran, C. Cardin, M. Convery, V. Quillet and C. Harding, *J. Chem. Soc., Chem. Commun.*, 2463 (1994).
10 J. A. Castro, J. Romero, J. A. Garcia-Vasquez, A. Castineiras, A. Sousa and J. Zubieta, *Polyhedron*, **14**, 2841 (1995).
11 Jianmin Li, Huidong Chen, Qiangjin Wu, Xintao Wu, *Cryst. Res. and Technol.*, **28**, 181 (1993).
12 Yu-Peng Tian, Chun-Ying Duan, Xue-Xiang Xu and Xiao-Zeng You, *Acta Cryst.*, **C51**, 2309 (1995).
13 R. Baggio, S. Baggio, M. J. Pardo and M. T. Garland, *Acta Cryst.*, **C52**, 820 (1966).
14 M. Nardelli, G. Fava and G. Giraldi, *Acta Cryst.*, **15**, 227 (1962).
15 S. Baggio, R. F. Baggio and P. K. de Perazzo, *Acta Cryst.*, **B30**, 2166 (1974).
16 R. Baggio, S. Baggio, M. I. Pardo and M. T. Garland, *Acta Cryst.*, **C52**, 1939 (1996).
17 R. Baggio, M. I. Pardo, S. Baggio and M. T. Garland, *Acta Cryst.*, **C53**, 727 (1997).
18 K. Nakamoto, *Infrared and Raman Spectra of Inorganic and Coordination Compounds*; Wiley, New York, (1986)
19 A. B. P. Lever, "*Inorganic Electronic Spectroscopy*", Elsevier, Amsterdam, London, New York, (1984).
20 Anonymous (1995) Recueil de normes françaises Antiseptiques et désinfectants .Afnor ed, Paris, France.
21 R. A. Mac Leod. J. Bliol. Chem., **197**, 751 (1952).
22 A. Altomare, G. Cascarano, G. Giacovazzo, A. Guagliardi, M. C. Burla, G. Polidori and M. Camalli, *J. Appl. Cryst.*, **27**, 435 (1994).
23 E. Prince. *Mathematical Techniques in Crystallography*, Berlin, Springer-Verlag, (1982).
24 D. J. Watkin, C. K Prout, J. R. Carruthers and P. W. Betteridge. *CRYSTALS Issue 10*, Chemical Crystallography Laboratory, University of Oxford, Oxford, (1996).
25 D. J. Watkin, C. K. Prout, L. J. Pearce. *CAMERON*, Chemical Crystallography Laboratory, University of Oxford, Oxford, (1996).
26 *International Tables for X-ray Crystallography*, Kynock Press, Birmingham, England, Vol IV, pp. 99 (1974).

**Received: June 23, 1998 - Accepted: July 10, 1998 -**
**Received in revised camera-ready format: October 15, 1998**

